# Design, Implementation, and Characterization of a Signal Acquisition Chain for SADino: The Precursor of the Italian Low-Frequency Telescope Named the Sardinia Aperture Array Demonstrator (SAAD)

**DOI:** 10.3390/s23229151

**Published:** 2023-11-13

**Authors:** Adelaide Ladu, Luca Schirru, Mauro Pili, Gian Paolo Vargiu, Francesco Gaudiomonte, Federico Perini, Andrea Melis, Raimondo Concu, Matteo Murgia

**Affiliations:** 1 National Institute for Astrophysics (INAF), Cagliari Astronomical Observatory, Via della Scienza 5, 09047 Selargius, Italy; adelaide.ladu@inaf.it (A.L.); mauro.pili@inaf.it (M.P.); gianpaolo.vargiu@inaf.it (G.P.V.); francesco.gaudiomonte@inaf.it (F.G.); andrea.melis@inaf.it (A.M.); raimondo.concu@inaf.it (R.C.); matteo.murgia@inaf.it (M.M.); 2 National Institute for Astrophysics (INAF), Istituto di Radioastronomia (IRA), Via Fiorentina 3513, 40059 Medicina, Italy; federico.perini@inaf.it

**Keywords:** microwave components for radio astronomy, low-frequency aperture array, radio frequency interference (RFI), ultra-high frequency (UHF), radio telescope, radio astronomy

## Abstract

Low-frequency aperture arrays represent sensitive instruments to detect signals from radio astronomic sources situated in the universe. In Italy, the Sardinia Aperture Array Demonstrator (SAAD) consists of an ongoing project of the Italian National Institute for Astrophysics (INAF) aimed to install an aperture array constituted of 128 dual-polarized Vivaldi antennas at the Sardinia Radio Telescope (SRT) site. The originally envisaged 128 elements of SAAD were re-scoped to the 16 elements of its precursor named SADino, with the aim to quickly test the system with a digital beam-former based on the Italian Tile Processing Module (iTPM) digital back-end. A preliminary measurements campaign of radio frequency interference (RFI) was performed to survey the less contaminated spectral region. The results of these measurements permitted the establishment of the technical requirements for receiving a chain for the SADino telescope. In this paper, the design, implementation, and characterization of this signal acquisition chain are proposed. The operative frequency window of SAAD and its precursor, SADino, sweeps from 260 MHz to 420 MHz, which appears very attractive for radio astronomy applications and radar observation in space and surveillance awareness (SSA) activities.

## 1. Introduction

Radio telescopes are useful instruments to observe the naturally occurring signals (i.e., radio waves) generated by all radio astronomic sources situated in the universe, such as stars, comets, planets, galaxies, and giant clouds of gas and dust [1]. Recently, radio astronomy has traditionally been focused on high-frequency observations [2], although it began at frequencies lower than 1 GHz. However, the potential of low frequencies has not been shelved in that it offers precious contributions for research activities concerning cosmic magnetism [3,4,5], epoch of reionization [6,7], high energy cosmic rays [8,9], solar and space weather [10], deep surveys of the radio sky [11], and the search for and characterization of transients and pulsars [12,13]. In the past decade, there has been a resurgence of interest in the field of low frequencies, and several types of radio telescopes have been designed and developed worldwide. Although their facilities and capabilities depend strictly on the operative frequency, these kinds of telescopes can be categorized into two main groups: aperture array systems and single-dish (or reflector) antennas. The basic principle of a phased array consists of orienting the maximum radiation in any desired angular sector by controlling the phase excitation between its elements using dedicated digital or analog phase shifters [14,15].

Several low-frequency aperture arrays have been implemented worldwide for radio astronomy applications [16]. Each of them is equipped with a dedicated signal acquisition chain, typically composed of microwave and radio frequency components such as switches, filters, low noise amplifiers (LNAs), and step attenuators. The receiving chain is designed specifically to reject the main radio frequency interference (RFI) and to take the detected signal to the digital back-end, which elaborates the data and beamforms them. Several portions of the frequency spectrum are covered by these instruments depending on the RFI scenario at the site. The Low-Frequency Array (LOFAR), the core of which is located about 3 km from the town of Exloo in the Dutch north-east, can be considered the largest radio telescope of the world that combines signals from tens of stations, distributed in Germany, France, Sweden, and the United Kingdom [17,18,19,20,21]. In particular, LOFAR consists of 48 stations, each of which has 48 or 96 antenna elements and is optimized for a bandwidth of 10–240 MHz. 

Further details about its antenna design and receiving chain are reported in [17,18,19,20,21]. The Long Wavelength Array (LWA) is located in central New Mexico and represents another radio telescope that utilizes relatively low frequencies (i.e., 10–88 MHz). The project involves the installation of 53 phased array stations, each of which is composed of 256 pairs of dipole elements distributed over a region about 400 km in diameter. Its front-end technical features are described in detail in [22,23]. The Murchison Widefield Array (MWA), implemented at the Murchision Radio-astronomy Observatory (MRO) in the Murchison region of Western Australia, consists of a dipole-based aperture array synthesis that works in the frequency range between 80 MHz and 300 MHz. It is composed of 128 tiles of 16 elements each, which is equipped with a dedicated analog beam-former, a receiver node for the signal digitalization and the bandwidth selection, a correlator for the cross-correlations of all signal pairs, and, finally, the real-time computer for the elaboration [24,25,26]. It represents a precursor for the Square Kilometre Array (SKA) as it concerns the low radio frequencies (SKA-low). The SKA-low project, which will stretch from the Murchision region to the Wajarrai Yamaji lands (Australia), will be composed of 512 stations of about 40-m diameter each, each consisting of 256 log-periodic dual-polarized antennas and will operate in the low-frequency range (50–350 MHz) of the SKA bandwidth. From a central compact core measuring 1 km across, further stations will be distributed in three spiral arms radiating from the center across a huge area; the maximum distance between the two furthest stations will be about 65 km. A detailed description of the technical features of the antennas and the SKA-low signal acquisition chain is reported in [27,28,29,30]. The Aperture Array Verification System 2 (AAVS2), operational since late 2019, represents the last full-size engineering prototype station (i.e., 256 elements pseudo-randomly distributed over a circular area of about 40-m diameter) deployed at the MRO site before the start of the SKA-Low construction phase. The characterization of its performance through commissioning observations is reported in [31].

In Italy, the Northern Cross Radio Telescope represents a large transit telescope that operates at a central frequency of 408 MHz with a 16-MHz bandwidth. The telescope is T-shaped and consists of an East-West arm (single reflector composed of 1536 dipole elements) and a North-South arm (composed of 4096 dipole elements). The signal acquisition chain is designed to down-convert the radio frequency signal to an intermediate frequency of 30 MHz. Accurate details and performance of this instrument are presented in [32,33,34].

An Italian ongoing development is represented by the Sardinia Aperture Array Demonstrator (SAAD), a project of the Italian National Institute for Astrophysics (INAF) aimed to install an aperture array constituted by 128 dual-polarized Vivaldi antennas at the Sardinia Radio Telescope (SRT) site [35]. In the first phase of the study, the covered frequency range sweeps from 50 to 500 MHz for observations of the sky at low radio frequencies. The objective of SAAD was oriented towards offering a technological and scientific test bed to gain experience and apply algorithms and techniques for digital beamforming, instrument calibration, imaging, and RFI mitigation. In addition, given the closeness with SRT, joined experiments correlating SAAD with a single-dish system such as SRT will represent an important attraction for researchers. Design, electromagnetic simulations, and characterization of the Vivaldi antenna prototype selected for the SAAD telescope are available in the literature [36,37,38,39]. The originally envisaged 128 elements of SAAD were re-scoped to 16 elements of SADino, with the aim to quickly test the system with a digital beam-former based on the Italian Tile Processing Module (iTPM) back-end [40]. Consequently, SADino can be defined as a precursor of the SAAD telescope. The iTPM board is based on two Field Programmable Gate Array (FPGA) boards capable of digitizing and processing up to 32 radio frequency input signals with a maximum bandwidth of 500 MHz [41].

In this paper, the status of the SADino project is proposed. The design, implementation, and characterization of a signal acquisition chain for SADino are accurately described. Unfortunately, the RFI scenario at low frequencies is unfavorable for radio astronomy research at the telescope site. An RFI measurements campaign is, therefore, necessary to have an idea of the spectral occupancy at low frequencies and select the less contaminated spectral region for SADino (and SAAD, consequently). On the basis of these considerations, the technical features of the microwave components, which compose the receiving chain, can be accurately established. The results of a preliminary RFI measurements campaign are discussed in Section 2. These results are useful for the design of an ad hoc signal acquisition chain for the SADino telescope, which is described in Section 3. The results of a laboratory characterization and the first spectrum acquired using the new receiving chain with an antenna installed at the SRT site are reported and analyzed in Section 4. In Section 5, conclusions about the work and the future perspectives are finally presented.

## 2. Preliminary Measurements Campaign of Radio Frequency Interference

**Figure 1:**
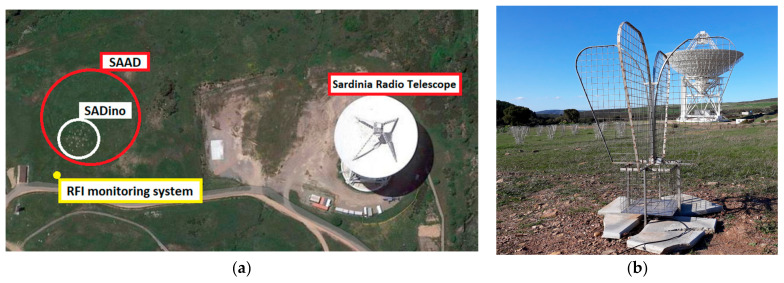
(**a**) Map of the Sardinia Radio Telescope (SRT) site where the SAAD telescope, its precursor named SADino, and the system for the monitoring of radio frequency interference (RFI) are installed; (**b**) photo of one of the SAAD’s Vivaldi antennas with SRT in the background.

Preliminary low-frequency RFI investigations at the SRT site were conducted in 2020 using a system composed of one of the SAAD’s Vivaldi antennas directly connected to a receiving chain of radio frequency components for the transport of the signal to the back end, which was based on a spectrum analyzer. A map of the SRT site, with the position of the SAAD and SADino’s antennas and the RFI monitoring system, is depicted in Figure 1.

The receiving chain of the RFI monitoring system is composed of two LNAs, one for each polarization channel, directly connected to the Vivaldi antenna. After that, a power combiner is used to merge the two polarization channels since this system aims to map the entire RFI scenario, including any signal. The power combiner is connected to a bias-tee through a coaxial cable. This component provides the power supply for the two LNAs. Finally, the receiving chain ends with the connection through a 20-m coaxial cable to the spectrum analyzer. Further details on the features of each component of this receiving chain are reported in [42]. The spectrum analyzer is remotely controlled by an ad hoc homemade LabView software that permits setting its parameters, such as resolution bandwidth (RBW), start and stop frequencies of the observation window, and duration of the temporal acquisition window, etc. [42]. The RFI monitoring system permits the detection of both continuous and impulsive signals. These two kinds of signals can be discriminated, setting a particular value for the RBW parameter of the spectrum analyzer. In more detail, the detection of continuous signals can be achieved with an RBW approximately equal to a few kHz (i.e., 100 kHz), while the survey of impulsive signals demands a higher RBW, such as a few MHz (i.e., 1 MHz).

**Figure 2:**
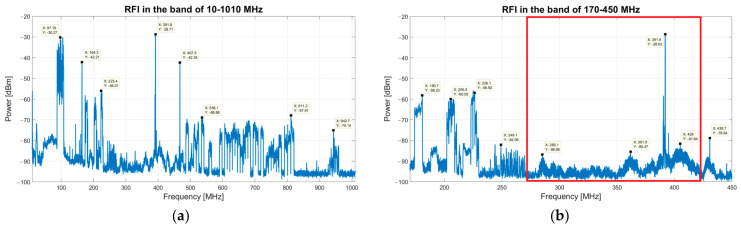
(**a**) Results of the RFI measurements campaign in the frequency range between 10 MHz and 1010 MHz. (**b**) Results of the RFI measurements campaign in the band of 170–450 MHz. The less contaminated spectral region is highlighted in red and sweeps from 260 MHz to 420 MHz.

The RFI investigation starts by considering a wide bandwidth between 10 MHz and 1010 MHz, then it decreases to two gradually narrower bands such as 110–500 MHz and 170–450 MHz. This band reduction is based on a rejection of the portions with a high density of RFI. An acquisition time window of two weeks is considered for each frequency band. The results of these measurements are summarized in the two plots of Figure 2. Each plot includes both continuous and impulsive signals, which are merged to provide a compact graphical representation.

Analyzing the band of 10–1010 MHz (see Figure 2a), the highest permanent signals belong to the frequency modulation (FM) radio services (i.e., 80–110 MHz), radio TV links (470–828 MHz), Sardinia emergency department communications (460 MHz) and mobile communications (900–950 MHz) [35,43,44]. Reducing the investigation bandwidth to 170–450 MHz (see Figure 2b), the main signals with high levels of amplitude derive from security services (160–185 MHz) and digital video broadcasting—terrestrial (DVB-T) services (177.5–226.5 MHz) [35,43]. Another signal with very high levels of amplitude can be clearly observed in both graphs at 385–395 MHz. This signal, recently studied with a dedicated measurements campaign [35], derives from the Terrestrial Trunked Radio (TETRA) system for radio communications of the Italian Ministry of Defense. Unfortunately, one of the many TETRA stations is located on the Monte Ixi Mountain, in a beeline only 1 km from the SRT site.

Finally, the band of 260–420 MHz, highlighted in red in Figure 2b, seems to be the less contaminated spectral region at less than the strong TETRA signals. This range can be considered as the operative frequency window of the SAAD telescope and its precursor SADino, which appears to be very attractive for radio astronomy applications [3,4,5,6,7,8,9,10,11,12,13,45] and radar observation of resident space objects in space and surveillance awareness (SSA) activities [46,47,48,49].

In addition to these results, a further RFI measurements campaign was recently performed using an RFI mobile laboratory owned by INAF [50]. These measurements, dating from the end of 2022 to early 2023, were useful in mapping the RFI scenario around the SRT site with the aim of redefining the observation sub-bands of the coaxial L-P cryogenic receiver of SRT. This recent work was focused on the frequency band of 250–450 MHz, and the 2020 results obtained from the campaign for SADino, presented in this paper, were generally confirmed.

## 3. Design and Implementation of a Signal Acquisition Chain for SADino

**Figure 3:**
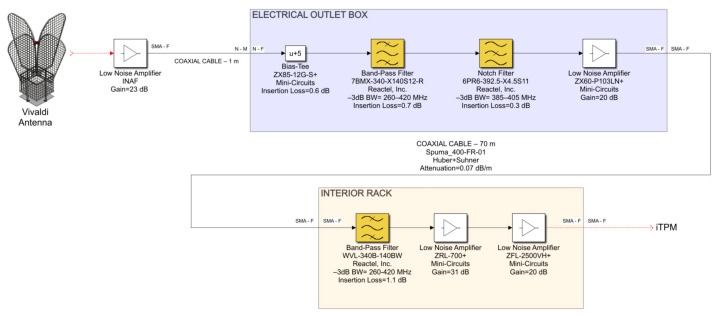
Schematic of the new signal acquisition chain of microwave and radio frequency components for the SADino system.

The results of the RFI investigation presented in Section 2 are fundamental to establishing the technical specifications for the microwave and radio frequency components that compose the dedicated receiving chain for SADino. The frequency window selected for SADino sweeps between 260 MHz and 420 MHz. The technical features of the receiving chain components are chosen to reject the strong RFI outside that range, which is still detected by the Vivaldi antenna in its operating frequency band. A detailed schematic of the signal acquisition chain is reported in Figure 3. It is composed of a Vivaldi antenna of the SAAD system (described in detail in Section 3.1), directly connected to a LNA developed by INAF engineers (Section 3.2). Each antenna is also equipped with an electrical outlet box where a bias-tee, two microwave filters, and an LNA are installed. This box is accurately described in Section 3.3. Each electrical outlet box is directly connected through a 70-m coaxial cable (Section 3.4) to an interior rack (Section 3.5), in which another microwave filter and two further amplification stages are installed.

This receiving chain is used for each of the two polarization channels (i.e., X and Y polarization) of each of the 16 Vivaldi antennas. Consequently, 32 identical receiving chains were implemented for the entire SADino system.

### 3.1. Vivaldi Antenna

The Vivaldi antenna prototype is designed by the Italian team for the project named Square Kilometer Array—Low-Frequency Aperture Array (SKA-LFAA) [51] and proposed for the SAAD telescope in its version 3.1 (see Figure 1b). The main technical and electromagnetic characteristics are listed in the following:

- Operating frequency range: the antenna prototype was developed to operate in a wide frequency range from 50 MHz to 500 MHz [36,37,38,39];
- High flexibility: the mechanical design consists of a mesh steel structure of 16 kg and size of 962 × 962 × 1370 mm. This design guarantees wind resistance with speeds up to 130 km/h. The antenna is fixed to the soil using four tiles (500 × 500 × 40 mm and 21 kg each) at its base in order to guarantee enough robustness and stability. By removing the tiles, the array configuration can be easily changed [36,37,38,39];
- Half power beam width (HPBW) of approximately 45 degrees: the radiation pattern of the Vivaldi antenna was characterized with a UAV-based measurement system, showing an antenna gain at the zenith of 7.8 dBi at 250 MHz and 8.6 dBi at 450 MHz [39];
- Unbalanced 50-ohm excitation through the use of two coaxial cables directly connected to the radiators: the inner part of the coaxial connector is linked to one wing, while the external one is embedded in the opposite wing. In this manner, coupled currents on the cable are not present;
- Linear dual-polarization (i.e., X and Y polarizations);
- Low cross-polarization on the principal axes: each antenna is equipped with a cubic cavity under the four wings to reduce the back lobes and improve the directivity and sensitivity;
- Reflection coefficient (S11) of about −9 dB in the frequency band of 250–450 MHz [39].

### 3.2. INAF Low Noise Amplifier

**Figure 4:**
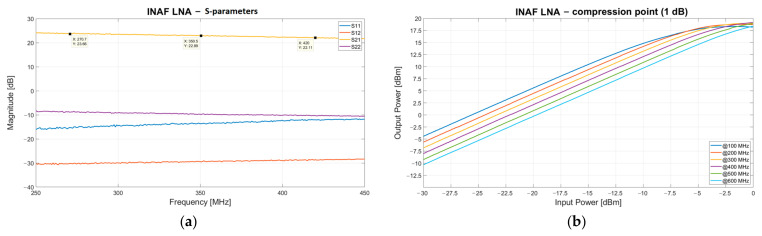
(**a**) S-parameters of the low noise amplifier (LNA) designed and implemented by engineers from the Italian National Institute for Astrophysics (INAF). (**b**) Output 1 dB compression point (OIP 1 dB) of the INAF LNA.

The model of LNA directly connected to the Vivaldi antenna is designed and implemented by engineers from INAF. The device is placed inside a metallic case that guarantees sufficient protection from atmospheric agents. A LNA is located in the bottom part of each antenna wing (one LNA for each polarization channel of each Vivaldi antenna), close to the probes. In detail, the device consists of a one-stage 50 Ω single-ended LNA based on a Monolithic Microwave Integrated Circuit (MMIC) amplifier (SPF-5122Z model from Qorvo) [52,53]. The LNA biasing is supplied through the coaxial cable attached to the output connector, and for this reason, a bias-tee in the acquisition chain is needed. The LNA presents a typical gain of up to 23 dB in the frequency range of 250–450 MHz (see Figure 4a), a noise figure of 0.5 dB, and a 1 dB compression point (IP1dB) up to −3 dBm (see Figure 4b).

### 3.3. Electrical Outlet Box

**Figure 5:**
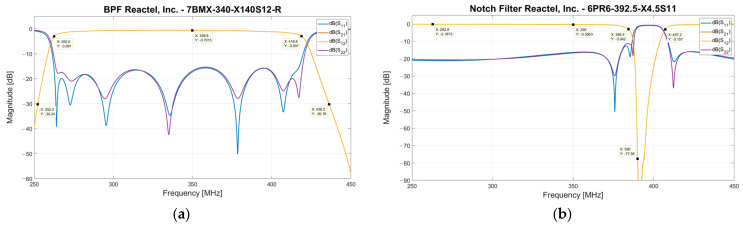
(**a**) S-parameters of the bandpass filter (BPF) from Reactel, Inc., model 7BMX-340-X140S12. (**b**) S-parameters of the Notch filter from Reactel, Inc., model 6PR6-392.5-X4.5S11.

Each INAF LNA is directly connected, using a 1-m coaxial cable, to an electrical outlet box (see Figure 3), which is installed on the bottom of the Vivaldi antenna. Each box is equipped with two identical portions of the receiving chain, which are useful for each polarization channel of the antenna (i.e., X and Y polarization). A bias-tee (model ZX85-12G-S+ from Mini-Circuits, New York, NY, USA [54]) with a low insertion loss (approximately 0.6 dB) useful to bias the INAF LNA is the first component of each electrical outlet box. The following components are the two highest-performing microwave filters of the entire receiving chain. The first filter is a band pass filter (BPF), model 7BMX-340-X140S12 from Reactel, Inc., Gaithersburg, MD, USA [55], which has a −3 dB band between 260 MHz and 420 MHz and an insertion loss of approximately 0.7 dB (see S21 parameter of Figure 5a). It is a seven-pole filter that provides an edge band with a very steep drop, such as –30 dB at 250 MHz and 436 MHz. In cascade with this BPF filter, a Notch filter (model 6PR6-392.5-X4.5 S11 from Reactel, Inc., Gaithersburg, MD, USA [55]) is installed for the rejection of TETRA signals at 385–395 MHz. This filter offers an insertion loss of approximately 0.3 dB and an attenuation of about 70 dB at the frequency range of interest (see Figure 5b), which guarantees a total cut of the aforementioned unwanted signals.

The last component installed in the electrical outlet box is an LNA (model ZX60-P103LN+ from Mini-Circuits, New York, NY, USA [56]), which represents the second amplification stage of the entire receiving chain. This active component provides a gain of up to 20 dB and a noise figure of about 0.5 dB. It needs to be powered with a DC supply voltage of 5 V and a current of 95 mA [56].

**Figure 6:**
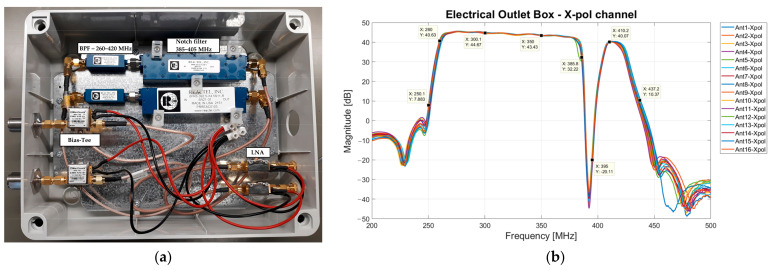
(**a**) Photo of the electrical outlet box with its microwave components. (**b**) Total gain of the portion of the receiving chain installed in the electrical outlet box.

All microwave components of the electrical outlet box are equipped with Sub-Miniature Version A (SMA) connectors, as shown in Figure 6a. The DC supply necessary to power the active components (i.e., the Bias-Tee and the LNA) is provided by a dedicated linear power supply, model HB5-3/OVP-AG (5 V, 3 A) from Bel Power Solutions, Inc., New York, NY, USA [57].

For each X-pol channel of each antenna, the total gain of this portion of the receiving chain is plotted in Figure 6b. The −3 dB band sweeps between 260 MHz and 420 MHz with a gain of approximately 44 dB, and the attenuation at the range of 385–395 MHz is approximately from 10 dB to 70 dB. Finally, there is an attenuation of about 30 dB at the frequency extremes, such as 250 MHz and 435 MHz.

### 3.4. 70-m Coaxial Cable

**Figure 7:**
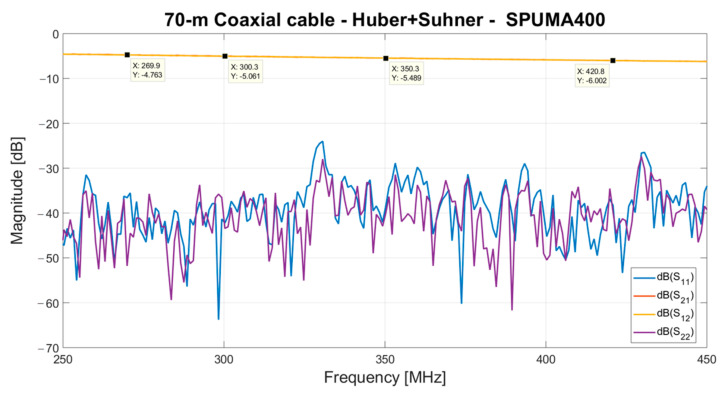
S-parameters of the 70-m coaxial cable from Huber + Suhner, model Spuma_400-FR-01.

Each Vivaldi antenna, with its electrical outlet box, is directly connected to the control room, where a rack cabinet with the iTPM digital back-end is placed. The distance between the array of Vivaldi antennas and the control room is approximately 70 m. Consequently, a 70-m flexible coaxial cable (model Spuma_400-FR-01 from Huber + Suhner, Charlotte, NC, USA [58]) is considered to cover this path in the receiving chain. It is characterized by a low attenuation of approximately 5.5 dB, in accordance with the specifications of its datasheet [58]. The S-parameters of the cable are plotted in Figure 7.

### 3.5. Interior Rack

A rack cabinet is placed in the control room to host the digital back-end based on the iTPM board. Since it requires a high level of power density at its input to guarantee its optimal functioning [40], two additional amplification stages are necessary for the receiving chain. For this reason, a rack composed of 8 modules, known as the interior rack, is implemented. Each module is equipped with four channels (one module serves two antennas, including their two polarization channels), each of which is based on a BPF and two LNAs.

**Figure 8:**
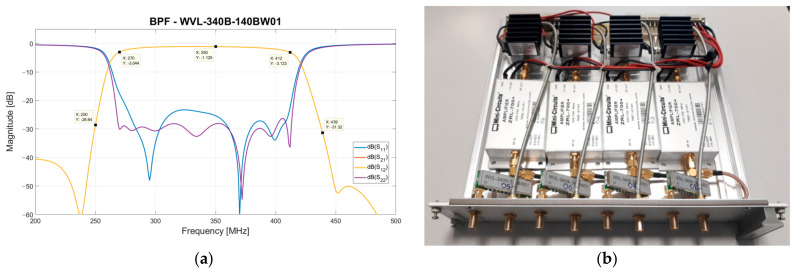
(**a**) S-parameters of the BPF (model WVL-340B-140BW01 from Wevercomm, Suwon, Republic of Korea) installed in the interior rack. (**b**) Photo of one of the eight modules of the interior rack. Each module is equipped with four identical channels for serving two dual-polarized antennas.

The BPF (model WVL-340B-140BW01 from Wevercomm, Suwon, Republic of Korea [59]) has a −3 dB band (i.e., 270–415 MHz) similar to the BPF (model 7BMX-340-X140S12 from Reactel, Inc., Gaithersburg, MD, USA) installed in the electrical outlet box. Its general performance is lower than that of the BPF of the electrical outlet box, offering an insertion loss of about 1.1 dB and lower slopes at the edges of its frequency response (see its S-parameters in Figure 8a). This filter has the role of emphasizing the frequency band of SADino after a path of the receiving chain of about 70 m.

After the BPF, the third LNA (model ZRL-700+ from Mini-Circuits, New York, NY, USA [60]) of the entire receiving chain is installed. It is characterized by a typical gain of 30 dB and a typical noise figure of 2 dB. It needs to be powered with a DC supply voltage of 12 V and current of 450 mA [60], provided by a dedicated linear power supply (model HE12-10.2-AG (12 V, 10.2 A) from Bel Power Solutions, Inc., New York, NY, USA [61]).

Finally, the last LNA of the chain (model ZFL-2500VH+ from Mini-Circuits, New York, NY, USA [62]) has a gain of approximately 20 dB, a typical noise figure of 5.5 dB, and a DC supply voltage of 15 V (current of 300 mA). It is biased by a dedicated linear power supply (model F15-15-A+G (15 V, 15 A) from Condor—DC Power Supply [63]).

Even components of the interior rack are equipped with SMA connectors, and a photo of one module of the interior rack is shown in Figure 8b.

**Figure 9:**
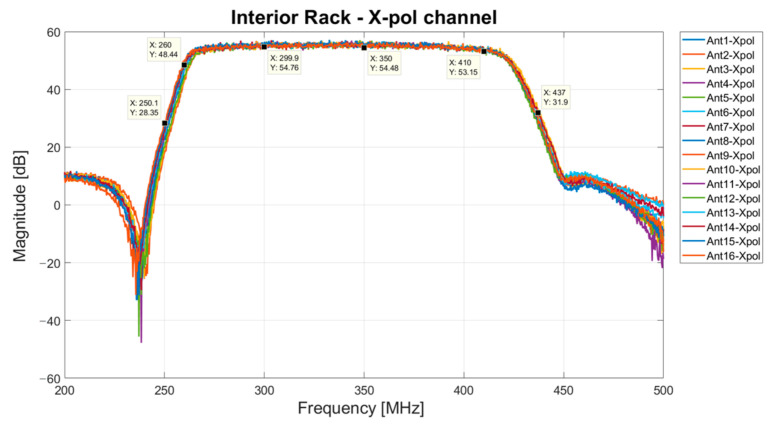
Total gain of the X-pol channels installed in the eight modules that compose the interior rack.

The total gain of each X-pol channel of the interior rack’s modules is graphed in Figure 9. The curve is similar to the frequency response of the BPF (model WVL-340B-140BW01 from Wevercomm, Suwon, Republic of Korea), with a gain of approximately 54 dB at the band of interest.

## 4. Results and Discussion: Laboratory Characterization and Real Spectrum Acquisition

A summary of the main features (maximum attenuation, gain, and noise figure) of all components of the receiving chain is reported in Table 1.

**Figure 10:**
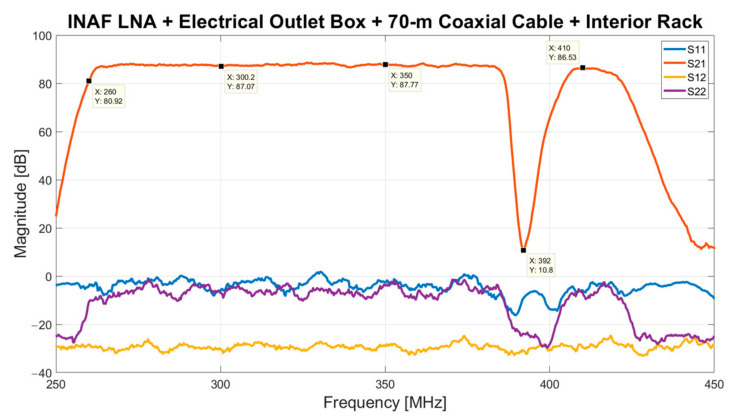
S-parameters of the entire receiving chain.

The performance of the entire signal acquisition chain is evaluated by analyzing its S-parameters, as shown in Figure 10. The red curve represents the S21 parameter, known as the gain of the chain. The optimization of the chain at the frequency band of 260–420 MHz is achieved. In detail, the measured total gain in this frequency window is typically 87 dB, in accordance with the estimated value of 85.8 dB achieved using the typical specifications summarized in Table 1. This order of magnitude is sufficient to fulfill the expected power of the input signal for the iTPM digital back-end [40]. The strong TETRA signals at 385–395 MHz are sufficiently rejected owing to an attenuation of approximately 70 dB.

In addition, an estimation of the total noise figure of the receiving chain can be calculated. Both the noise figure and gain values of Table 1 can be expressed as a noise factor (in linear, not in dB) and linear gain, respectively. The following Friis formula is used to calculate the total noise factor *F_tot_* of the receiving chain:

(1) $$F_{tot}=F_{1}+\frac{F_{2}-1}{G_{1}}+\frac{F_{3}-1}{G_{1}G_{2}}+\frac{F_{4}-1}{G_{1}G_{2}G_{3}}$$

where *F* and *G* represent the noise factor and the gain of each LNA of the chain, respectively. The total noise factor *F_tot_* of the SADino receiving chain is equal to 1.12, which can be expressed as a noise figure of approximately 0.5 dB. In addition, the noise figure of the receiving chain can be equivalently expressed in terms of noise temperature *T_REC_*, using the following formula:

(2) $$T_{REC}=290\cdot (F_{tot}-1)$$

where 290 is the ambient temperature expressed in Kelvin, and *F_tot_* represents the total noise factor previously calculated. The noise temperature *T_REC_* of each channel of SADino is approximately equal to 34.8 K.

Finally, the total system noise temperature *T_SYS_* can be estimated for the worst case at 400 MHz using the following equations:

(3) $$\begin{array}{c} T_{SYS}=T_{a}+T_{REC}\\ \mathrm{where}\;T_{a}=T_{SKY}+T_{GROUND} \end{array}$$

where *T_A_* represents the antenna temperature, *T_SKY_* is the sky temperature approximately equal to 30 K at 400 MHz [64], and *T_GROUND_* consists of the noise contribution from the ground detected by the antenna, which is approximately equal to 25 K [38]. Considering these values, the estimation of the system noise temperature *T_SYS_* is equal to 89.8 K. This achievement is in line with the estimation and measurement of the noise system temperature of the Italian Northern Cross Radio Telescope at the same frequency [34].

**Figure 11:**
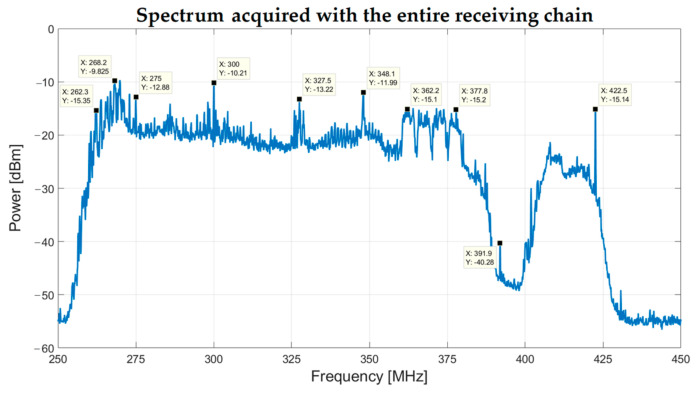
The spectrum between 250 MHz and 450 MHz was acquired using a spectrum analyzer as the back-end, one Vivaldi antenna installed on the laboratory rooftop, and its signal acquisition chain.

The entire signal acquisition chain is also tested using one of the 16 Vivaldi antennas of SADino, installed on the laboratory rooftop, and a spectrum analyzer as the back-end. The acquired spectrum is shown in Figure 11.

It is clear that the TETRA signals in the frequency range between 385 MHz and 395 MHz are completely rejected, with a level 30 dB lower than the noise floor of the SADino band. For this reason, a lower-performing and cheaper Notch filter can be considered in the future for the extension of SADino to the entire SAAD system of 128 elements.

Since for this test, the Vivaldi antenna was installed on the laboratory rooftop, some unwanted signals from the alongside air-conditioning system were detected at 260–300 MHz. These signals will not be supposedly received by the SADino system, which is installed away from the laboratory. However, if these signals from the air-conditioning system are detected by SADino, they will be greatly attenuated by the distance of the installation field from the RFI source. In addition, a signal from a narrowband military communications satellite system is clearly noticeable at 360–380 MHz. This signal does not represent a criticality for SADino because its amplitude level is not high enough to compromise the linear response of the receiving chain.

## 5. Conclusions and Future Work

In the developing phase of a radio telescope, preliminary RFI measurements are necessary to investigate the main unwanted signals and choose the less contaminated spectral region of the entire possible frequency window. In the case of SADino, the precursor of the Italian low-frequency telescope called SAAD, the frequency band between 260 MHz and 420 MHz is chosen. This band is relatively free of RFI with very high levels of amplitude unless there are strong signals from the TETRA system at 385–395 MHz. Since these signals can saturate the digital back-end and represent trouble for astronomy research, a receiving chain is developed in order to reject them. This signal acquisition chain is designed using microwave components optimized in the frequency range of 260–420 MHz. It is composed of four amplification stages and three microwave filters. In particular, the first amplification stage is provided by an LNA, directly connected to the Vivaldi antenna, with a low noise figure (i.e., 0.5 dB). The two main filters of the receiving chain are a BPF, which selects the entire bandwidth for SADino (260–420 MHz), and a Notch filter for the rejection of the unwanted signals from TETRA (attenuation of approximately 70 dB at 385–395 MHz). These two components, along with an LNA, are contained in an electrical outlet box installed at the base of each Vivaldi antenna of the SADino array (composed of 16 elements). Each element is connected to the control room through a 70-m coaxial cable characterized by low attenuation (approximately 5.5 dB). In the control room, a rack for each antenna, with two other LNAs and another BPF (260–420 MHz), is installed in the rack cabinet that hosts the iTPM digital back-end. The entire receiving chain has a typical gain of about 87 dB, a noise figure of about 0.5 dB, and a noise temperature of approximately 34.8 K. The noise system temperature for the SADino system can be consequently estimated and results equal to 89.8 K. This achievement is in line with the estimation and measurement of the noise system temperature of a similar telescope that operates at the same frequency (the Italian Northern Cross Radio Telescope).

After the design, implementation, and laboratory characterization of the receiving chain proposed in this paper, its field installation will be performed. In this way, each of the 16 Vivaldi antennas of SADino will be equipped with its signal acquisition chain that connects it to the iTPM digital back-end. After that, the first light of the SADino telescope will be finally executed, testing the level signals at the back-end input and performing digital beamforming operations.

## Figures and Tables

**Table 1:** Summary of the attenuation, gain, and noise figure values of the microwave components that compose the receiving chain.

Receiving Chain Components	Max Attenuation [dB]	Gain [dB]	Noise Figure [dB]
INAF LNA	–	23	0.5
Bias-Tee, ZX85-12G-S+, Mini-Circuits	0.6	–	–
BPF, 7BMX-340-X140S12-R, Reactel, Inc.	0.7	–	–
Notch filter, 6PR6-392.5-X4.5S11, Reactel, Inc.	0.3	–	–
LNA, ZX60-P103LN+, Mini-circuits	–	20	0.5
70-m coaxial cable, Spuma_400-FR-01, Huber+Suhner	5.5	–	–
BPF, WVL-340B-140BW01, Wevercomm	1.1	–	–
LNA, ZRL-700+, Mini-circuits	–	31	2
LNA, ZFL-2500VH+, Mini-circuits	–	20	5.5

## Data Availability

Data is contained within the article.
